# Viral Infections in Burn Patients: A State-Of-The-Art Review

**DOI:** 10.3390/v12111315

**Published:** 2020-11-17

**Authors:** Jacek Baj, Izabela Korona-Głowniak, Grzegorz Buszewicz, Alicja Forma, Monika Sitarz, Grzegorz Teresiński

**Affiliations:** 1 Chair and Department of Human Anatomy, Medical University of Lublin, 20-090 Lublin, Poland; 2 Department of Pharmaceutical Microbiology with the Laboratory of Microbiological Diagnostics, Medical University of Lublin, 20-090 Lublin, Poland; iza.glowniak@umlub.pl; 3 Chair and Department of Forensic Medicine, Medical University of Lublin, 20-090 Lublin, Poland; g.buszewicz@umlub.pl (G.B.); aforma@onet.pl (A.F.); grzegorzteresinski@umlub.pl (G.T.); 4 Department of Conservative Dentistry with Endodontics, Medical University of Lublin, 20-090 Lublin, Poland; mksitarz@gmail.com

**Keywords:** burn, herpesvirus, herpes simplex virus, cytomegalovirus, varicella zoster virus, human immunodeficiency virus

## Abstract

Infections that are triggered by the accompanying immunosuppression in patients with burn wounds are very common regardless of age. Among burn patients, the most frequently diagnosed infections include the bacterial ones primarily caused by *Pseudomonas aeruginosa* or *Klebsiella pneumonia*, as well as fungal infections with the etiology of *Candida spp*. or *Aspergillus* spp. Besides, burn wounds are highly susceptible to viral infections mainly due to the impaired immune responses and defective functions of the immune cells within the wound microenvironment. The most prevalent viruses that invade burn wounds include herpes simplex virus (HSV), cytomegalovirus (CMV), human papilloma virus (HPV), and varicella zoster virus (VZV). Likewise, less prevalent infections such as those caused by the orf virus or Epstein–Barr Virus (EBV) might also occur in immunosuppressed burn patients. Viral infections result in increased morbidity and mortality rates in severely burned patients. Additionally, a positive correlation between the hospitalization duration and the severity of the viral infection has been demonstrated. Viral infections trigger the occurrence of various complications, ranging from mild symptoms to even fatal incidents. Accurate detection of viral infection is of great clinical importance because of the possibility for a quicker introduction of proper treatment therapy and shortening of hospitalization time. The aim of this paper is to provide a comprehensive review of the literature and summarize the findings regarding the most common viral infections in immunosuppressed burn patients.

## 1. Introduction

Burn wounds are highly susceptible to various infections—bacterial and viral, as well as fungal—that might be exogenous, endogenous, or opportunistic [1]. According to the World Health Organization (WHO), the approximate number of deaths due to burn wounds is estimated at 265,000 per year, among which over 96% occur in low- and middle-income countries [2]. The occurrence of a burn injury in a patient significantly affects the immune responses, resulting in secondary immunodeficiency that is further associated with poorer clinical outcomes. The major alterations include the decreased activity of neutrophils and the excessive release of cytokines and growth factors that result in persistent inflammation.

Burns are classified according to a four-degree scale depending on their severity, depth, the extent of tissues that are burnt, the exact location on the body, and the general clinical outcome. A first-degree burn refers to a superficial burn that only involves the epidermis. A second-degree burn, as well as the epidermis, also includes the most superficial layers of the dermis and a third-degree burn involves both—the epidermis and the dermis—whereas a fourth-degree burn passes through all of the skin layers, reaching the underlying structures, including muscles and bones. Burns weaken the immune system of a host, resulting in a wide spectrum of clinical manifestations, as well as further complications during the hospitalization course.

Except for the opportunistic infections that might occur in immunosuppressed burn patients, reactivations of latent viral infections are also very prevalent. Such reactivations can manifest either systematically or be limited only to the closest area of a burn wound. Incidents of asymptomatic viral reactivation have also been described in the literature and they can be diagnosed with the antibody positivity. Viral infections usually do not concern patients without immunodeficiency.

During the first stages of an acute burn, the number of microorganisms within the burn wound is small; afterwards, the site of the eschar is gradually colonized primarily by Gram-negative bacteria. Thereafter, there is a significant increase in the number of infections that might appear within the burn wound with time, which is also due to the disrupted microflora, increasing the probability of opportunistic infections [3,4]. Moreover, burn patients are much more susceptible to a significant number of infections due to the impairments of the immune cells and the following defective immune responses [5]. What should be considered during the assessment of the severity of the infection are the co-infections and diseases that may result in substantial immunosuppression, leading to a higher susceptibility to other pathogens. What is also significant is the degree of a burn and its characteristics, such as the depth and the range of tissues occupied. The most common cause of death in both adult and pediatric burn patients is multi-organ failure due to sepsis, whereas one of the most prevalent complications includes dysfunction of the respiratory system [6,7,8].

It is crucial to understand the pathophysiology and specific processes that successively appear one after another during the treatment therapy; this is of great clinical importance since microbiological flora within the burn might differ throughout all of these processes. Both clinicians and patients should be aware that the quickest appropriate treatment of a burn significantly minimizes the potential infections within the wound. During treatment, any macroscopic changes should be immediately taken for microbiological analysis. This enables the choice of the most efficient therapy, allowing for more effective and quicker recovery of the burn patients.

Several resistant microorganisms have emerged which may also affect the severity of the wound infections among burn patients. The group of resistant microorganisms primarily includes such bacteria as methicillin-resistant *Staphylococcus aureus*, vancomycin-resistant *Enterococcus*, or *Pseudomonas*, along with such fungi as non-albicans *Candida spp*. with *Aspergillus* or *Fusarium spp.* [9,10,11,12,13,14,15]. Currently, *Pseudomonas aeruginosa* and *Klebsiella pneumonia* constitute the most common microorganisms that colonize burn wounds [16,17].

Even though a wide spectrum of bacteria constitutes the majority of infections within the burn wounds, viral infections are still highly prevalent. Besides, there are incidents of infections where several different viruses coexist, which usually leads to more severe clinical outcomes [18]. According to D’Avignon et al., in a 2009 study, from among the 97 autopsies performed, viral infections were responsible for the deaths of five patients in the studied group; unfortunately, the diagnosis of a viral infection was not made until the autopsy [19]. Nonetheless, due to the advancements in the medical field, including the appearance of various effective treatment strategies and improved diagnostic techniques, a significant decrease in the morbidity and mortality rates amongst burn patients has been reported [20].

## 2. Herpesviruses

### 2.1. Herpes Simplex Virus (HSV)

#### 2.1.1. HSV Characteristics

Herpes simplex viruses type 1 and 2 (HSV-1 and HSV-2) belong to the *Herpesviridae* family that constitutes a group of enveloped viruses containing a relatively large dsDNA genome and presenting a short reproductive cycle [21]. The prevalence of both HSV-1 and HSV-2 infections increases with a patient’s age [22]. Regarding HSV-1, its prevalence varies from 27% among the individuals aged 14–19 to 41.3%, 54.1%, and 59.7% in the following age groups—20–29, 30–39, and 40–49, respectively. A similar prevalence pattern concerns HSV-2 infection—from 0.8% among individuals aged 14–19 and even up to 21.2% in the group aged 40–49 [23]. HSV viruses have an ability to establish their latency within the sensory ganglia of the autonomic nervous system and, therefore, they are prone to reactivation even after a long period of silence [24]. The recurrence of HSV infection after its latency might be induced after internal stimuli, like stress, fatigue, or fever, and external ones including chemical, mechanical, and inflammatory factors [25]. HSV reactivation primarily occurs in adult patients who were previously exposed to HSV infection, whereas primary infections are more common in children and usually present a more aggressive course as well as a longer duration. Generally, HSV-1 is mainly associated with the oral infections, whereas HSV-2 is more prone to infect skin areas (especially within the genital area) primarily during a state of the lowered immunity, such as in patients with burn wounds who present significant impairments of the local immunity and the microflora [26,27].

#### 2.1.2. HSV Infection and Burn Wounds

Generally, HSV infections are most prevalent within the donor sites and not directly within the burn wound [28]. Besides, HSV infections might also occur within partial-thickness burns that have recently healed. Active HSV infections within a burn wound significantly prolong the recovery time and impair the healing process [29]. HSV superinfections most frequently occur within burns on the upper limbs and thorax and do not present a potential for visceral dissemination [30]. It was reported that the majority of herpetic infections related to burn wounds affect males, however, this is probably due to the higher percentage of male entry to burn units and institutions [31]. Furthermore, HSV seropositivity is present in more than 25% of patients with burn injuries [32]. HSV infections in burn patients might occur either by the reactivation of a latent infection due to the decreased immunity or by a primary, opportunistic infection.

HSV infection-related impairments are facilitated by the disturbed immunological reactions mainly within the subpopulation of the suppressor T lymphocytes; since this lymphocytic subpopulation appears approximately 1–3 weeks after an initial burn wound, it is also the time of the greatest susceptibility to the primary HSV infection. HSV also induces the downregulation of Toll-like receptor (TLR)-mediated nuclear factor-κB (NF-κB) cytokine production, which enhances further viral replication [33]. Subsequently, the percentage of potential bacterial infections is also significantly increased in such patients [34]. The abovementioned immune-related alterations also include a decreased number of T lymphocytes and lowered lymphocytic responsiveness to mitogenic and HSV antigenic stimuli, as well as a decreased release of interleukin-2 (IL-2), and abnormal antibody production [35].

**Figure 1:**
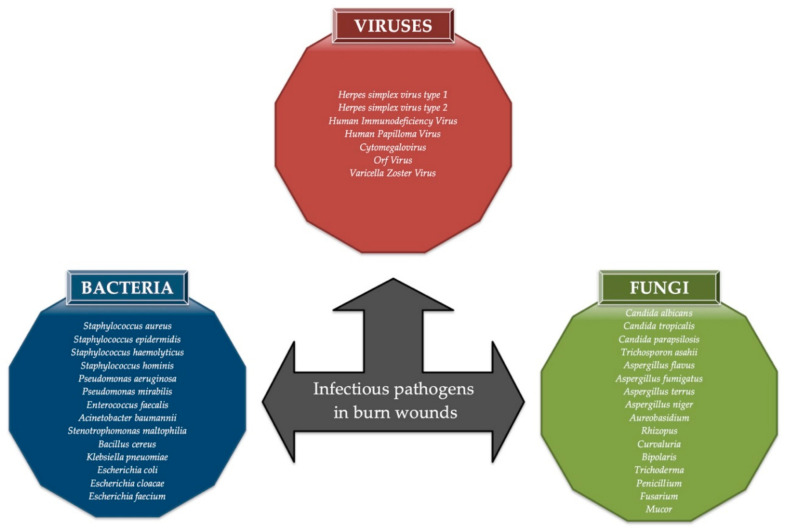
The examples of bacteria, viruses, and fungi that might induce infections within burn wounds.

Usually, HSV infections occur mainly within extensive burn wounds, whereas minor ones are less susceptible. Wurzer et al., in 2017 (Supplementary Table S1), found that HSV infections are quite prevalent in cases where the total body surface area (TBSA) of a burn is more than 53% [36]. However, unusual cases of HSV infection, when only a small percentage of the TBSA is affected by a burn wound, are still reported. Sobouti et al., in 2018, presented a case of a 1-year old infant whose TBSA was equal to 0.5%, but the HSV genome was still detectable via a polymerase chain reaction (PCR) and gene sequencing analyses [26]. Interestingly, in the aforementioned case, the number of the white blood cells was in the reference range and the TBSA percentage seemed to be too small to significantly lower the immunity responses in this individual’s case. Therefore, even when the percentage of the TBSA is relatively insignificant, clinicians should provide all of the essential examinations for bacterial, viral, and fungal infections to maximize the accuracy of the diagnosis (Figure 1) [37].

Besides, even small burn wounds may induce either the reactivation of latent infection or the induction of a primary infection. The severity of HSV infection is also unpredictable since it may vary from mild symptoms, such as vesicular skin rash, to even severe necrotizing hepatitis or fatal encephalitis [38,39,40,41].

#### 2.1.3. Clinical Manifestations of the HSV Infection in Patients with Burn Wounds

HSV infection manifests as a group of vesicles or vesicopustules within the area of a burn and usually occurs in the first three weeks after the injury. HSV reactivation might either appear within the area of a burn or manifest systematically. Besides, asymptomatic reactivation is also quite prevalent and must be verified and confirmed by the appropriate laboratory analyses. Facial or neck burns usually lead to the reactivation of HSV infection mainly because of the HSV latency within the trigeminal ganglion [42]. Regarding HSV-related diseases in burn patients, casual cases of tracheobronchitis along with pneumonia were reported [31]. Moreover, cutaneous infections can also be present but, nevertheless, they are not as common as the aforementioned medical conditions [43]. Kagan et al. showed that 52% of severely burned patients showed a significant increase in anti-HSV immunoglobulin G (IgG) titers to herpes viruses in general [44]. Fidler et al., in 2002, showed that approximately 2 weeks after the exposure of the burns within the head and neck areas, 15% of the intubated patients presented facial rash due to HSV infection [45]. Cook et al., in 2017, reported a case of a 58-year old woman with burn wounds who died because of diffuse HSV-related hepatitis and massive liver necrosis [46]. The last case required a liver biopsy, which constitutes a gold diagnostic standard for those patients who are suspected of HSV hepatitis; such incidents are relatively rare but might be fatal [47]. There was also a case in which HSV-2 infection was associated with severe pneumonitis, tracheitis, and focal necrotizing hepatitis in a severely burned patient [48]. Bordes et al. (2008) reported a case of a 43-year old man with a burn of 65% of the TBSA due to self-immolation. Because of the rapid state of unconsciousness, PCR tests were performed to investigate the potential cause of infection and the results showed HSV-1-related encephalitis, probably due to viral reactivation [49]. However, milder symptoms such as a rash, vesicles, or vesicopustules might be quite easily misdiagnosed and treated as impetiginous, eventually leading to much more severe complications of the HSV infection [50]. In 1996, Byers et al. demonstrated that 50% of burn patients showed the presence of HSV in the lung tissue and 16 patients developed acute respiratory distress syndrome (ARDS), among which 13 were infected by HSV [51]. Extremely severe complications of HSV infection in burn patients, such as hepatitis, liver necrosis, pneumonitis, tracheitis, encephalitis, or ARDS, are most likely due to the reactivation of a latent HSV infection, which may be induced by shock [52]. However, statistically, there is no correlation between active HSV infection and the increased morbidity and mortality rates in burn patients [53].

#### 2.1.4. HSV Detection and Treatment

The clinical diagnosis of HSV might be quite ambiguous usually due to the lack of specific symptoms. Regarding burn wounds, laboratory diagnostic methods should be applied even in cases of suspected viral infections because of the probability of asymptomatic HSV infection. Diagnostic inconsistency and inaccuracy might be due to the various methods of HSV detection, including the usage of serologic analysis, respiratory cultures of the virus, or even cutaneous or mucosal HSV cultures [29]. Regarding previously described cases, PCR and gene sequencing seem to be reliable methods for the detection of a viral infection. Currently, PCR constitutes the gold standard for the verification and diagnosis of HSV infection [54]. Besides, fluorescence in situ hybridization (FISH) and next-generation sequencing (NGS) are also applicable during HSV detection [55,56]. The presence of intranuclear eosinophilic inclusion bodies (Cowdry type A), that can be observed under a light microscope, provides a quick validation of the diagnosis. However, the presence of Cowdry type A bodies is also a characteristic of a varicella zoster virus (VZV) infection [57,58]. Even though some diagnostic techniques may vary and both false negative and false positive results should be taken into consideration, the number of HSV infections related to burn injuries is surprisingly high. The quickest and most accurate diagnosis of HSV infection provides a quicker introduction of the proper treatment therapy, enabling a reduction of the hospitalization course, potential complications, and re-infection episodes. The treatment of HSV infections usually includes acyclovir therapy but, nonetheless, alternative agents such as ganciclovir or foscarnet can also be applied [59,60]. As well as the traditional therapy, the potential role of l-lysine, an amino acid that interacts with arginine, essential for HSV replication, is currently being investigated [61].

### 2.2. Cytomegalovirus

#### 2.2.1. CMV Characteristics

Cytomegalovirus (CMV) is an enveloped virus that belongs to the *Herpesviridae* family and the *Betaherpesvirinae* subfamily. There are eight species currently known, with humans and monkeys acting as the natural hosts of the virus, but only one type—*Human betaherpesvirus 5* (CMV or HCMV)—is prone to infect humans, resulting in such diseases as mononucleosis or pneumonia [62,63]. Even though breastfeeding is a possible infection route, most CMV infections are assumed to be horizontally acquired community infections during the childhood period [64,65,66]. Primary human cytomegalovirus disease is defined as a combination of various clinical symptoms affecting different organs when CMV can be detected from the specific tissue or fluid samples [67]. The severity of CMV infections significantly differs—from mild symptoms or even symptomless cases—to severe diseases including pneumonia, encephalitis, hepatitis, colitis, or retinitis [68]. CMV disease can significantly affect the morbidity and mortality rates but, nevertheless, according to recent studies, CMV infection in burn patients does not directly affect those rates.

#### 2.2.2. CMV Infection and Burn Wounds

Traumas such as burn injuries significantly impair patients’ immune responses and, thus, increase the likelihood of developing a CMV infection. Adult patients with burn wounds may become affected by CMV either by the reactivation of latent infection or by a primary, exogenous infection. CMV latency occurs within the bone marrow, mainly within the monocyte/granulocyte progenitor cells [69]. Viral reactivation and further replication occur in the hematopoietic cells, mainly due to the internal and external stressors and stimuli [70].

The rate of a CMV reactivation in burn patients varies from 55% to 71% and, thus, constitutes a significant clinical concern [71]. The exact mechanism of CMV reactivation remains unclear but, nevertheless, it is believed that defective immune responses, including macrophage hyperactivity, enhanced cytokine production, and overactivation of type 2 T cells, might be crucial in this process [72,73]. A differentiation between the primary and reactivated CMV infection can be done with the usage of anti-CMV-IgG and microneutralization [74]. Due to the impaired immunological responses, including overactivation of T helper cells and defective responses from T cells, burn patients are more vulnerable to CMV reactivation [75]. The seroconversion rate of CMV in burn patients who were previously diagnosed as seronegative for CMV varies from 18% to 22% [76]. Moreover, up to 50% of patients with CMV latency can develop the reactivation of infection during immunosuppression. Regarding burn centers in Germany, 41% of the respondents claimed that CMV infection related to burn wounds is of significant clinical importance, whereas, in the United States, only 13% of the respondents believed in such a high relevance of CMV infections within burn lesions [77]. These results show that the awareness of CMV infections in burn patients may be questionable in some communities.

A direct relationship between CMV disease and bacterial sepsis has not been determined yet. However, CMV infection can significantly increase susceptibility to bacterial infections because of the decreased immune responses in infected individuals [78]. Bordes et al., in 2011, showed that CMV infection occurred mainly in CMV-seropositive burn patients with an incidence rate equal to 71%, whereas in the case of the seronegative burn patients, the infection rate was equal to 12.5% [79]. Patients with sepsis present nearly a five times higher percentage of CMV infections; recent studies also showed that latent CMV may be reactivated during intra-abdominal sepsis [80].

According to published reports, there is no correlation between the presence of CMV (neither reactivated nor primary) and increased mortality rates. CMV infections are most common in patients with burns greater than 15% of the TBSA. Hamprecht et al., in 2004, reported a case of a 40-year old female who suffered from burn injuries that were occupying approximately 65% of the TBSA [81]. The patient received skin transplants, including the areas of the arms and neck. Viral DNA and RNA were detected from the blood and lung samples, confirming the CMV presence; during the first day of the injury, CMV DNA was not detectable. CMV IgM titers were present in the serum approximately 25 days after a burn incidence, whereas the peak level was reported during the 39th day after a burn. CMV was detectable in the bronchoalveolar lavage (BAL) fluids despite a lack of manifestations from the respiratory system. This observation may be an explanation of the pulmonary distress that occurs even when no strict and relevant macroscopic changes are observed. In this case report, it was suspected that active CMV infection was due to the presence of the skin allografts, which could constitute a potential source of CMV vectors. Another more likely explanation for those findings might be a probable blood spillover of CMV.

CMV infection in burn patients may be induced due to the presence of CMV within skin allografts and further reactivation after transplantation in previously uninfected patients [81,82,83]. Gibbs et al., in 2016, presented a case of a burn patient who was reported to have developed severe colitis due to CMV infection [84]. CMV infection might result in numerous complications of varying severity, from symptomless cases of latency to major organ dysfunctions, including hepatitis, encephalitis, pneumonia, or colitis. One of the potential molecular mechanisms of CMV reactivation in cases of burn wounds is the polymorphism of human leukocyte antigen (HLA-E) loci [85]. As well as CMV infection alone, incidents of combined (CMV and HSV) infections in burn patients have been described [44,86].

#### 2.2.3. CMV Detection and Treatment

Diagnostic techniques of CMV detection include serological tests that reflect prior exposure to CMV, as well as other tests for active infection, such as PCR, conventional cell cultures, antigenemia, quantitative nucleic acid testing (QNAT), or immunochemistry [87]. The usage of PCR seems to be the most efficient method for potential CMV detection, enabling the quickest introduction of the appropriate treatment therapy. Histologic detection of CMV under a light microscope may be validated by the presence of intranuclear basophilic inclusion bodies with an “owl’s eye” appearance [88]. Currently, the most common serologic test used for measuring antibody to CMV is the enzyme-linked immunosorbent assay (ELISA) [89]. Antiviral therapy for CMV should be considerably adjusted for all patients, especially for those with renal dysfunction. The appropriate doses of antivirals should be provided since doses that are too low might induce antiviral resistance and overall treatment failure. Primarily, therapy includes ganciclovir, however, valganciclovir also constitutes an effective treatment agent [90].

### 2.3. Varicella Zoster Virus

#### 2.3.1. VZV Characteristics

Varicella zoster virus (VZV) is a human alphaherpesvirus that belongs to the *Herpesviridae* family. More than 90% of the population develops either primary infection, which manifests as chickenpox or shingles, or establishes a latent infection within the neurons of the trigeminal or dorsal root ganglia [91]. The reactivation of VZV occurs in approximately one-third of individuals with a latent infection and usually leads to shingles; post-herpetic neuralgia and delayed healing may also be caused by VZV reactivation [92,93]. The reactivation of VZV might be triggered by several stress factors, including other viral, bacterial, or fungal infections, trauma, immunosuppression, and lowered immunity [94].

#### 2.3.2. VZV Infection and Burn Wounds

VZV infections within burn injuries are extremely rare; nevertheless, whenever they occur, the post-infectious complications are usually critical and are associated with increased mortality rates [53]. Manifestations of VZV infection tend to be particularly severe mainly among patients with second-degree burns as well as within the healing donor sites [95]. The reactivation of latent VZV infections may be associated with the presence of extensive burn lesions and further impaired immunity. Likewise, donor sites present increased susceptibility to VZV infection [32]. The sources of infection typically include endogenous VZV or those which are induced by blood transfusions. Despite burn-related immunosuppression of patients, VZV reactivation is relatively rare in patients with burn wounds. Besides, the molecular trigger of VZV reactivation is yet unclear.

Sheridan et al. observed post-infectious cases of chickenpox and pneumonitis that were developed in pediatric burn patients with active VZV infection [86]. Moreover, in the same study, it was reported that the majority of burn patients who developed VZV infection were those who were previously exposed to the index case. It is still unexplained why the reactivation of HSV, which also belongs to the *Herpesviridae* family, is much more frequent when compared to VZV reactivation. VZV infections are highly prevalent in pediatric patients, especially those who were never affected by VZV or vaccinated [96]. The immunity to VZV, which is obtained either by a previous infection or VZV vaccination, significantly lowers the rate of VZV infections among burn patients [86]. Furthermore, morbidity and mortality rates are elevated among non-immunized patients with acute VZV infection.

#### 2.3.3. VZV Detection and Treatment

Laboratory diagnosis is crucial to differentiate between VZV and HSV infections since the clinical picture of VZV infection might be slightly confusing due to the dermatologic manifestations that are typical for HSV [97,98]. PCR is a sensitive, widely available technique that provides a rapid and accurate confirmation of a suspected VZV infection [99]. Sauerbrei et al. reported that PCR provides the highest rate of detected VZV infections (95%) comparing to culture (20%), serology (48%), or immunofluorescence (82%) [100]. Besides, light microscopy analysis reveals the presence of intranuclear inclusion bodies (Cowdry type A), which may also confirm the diagnosis [101]. Acyclovir remains the first-line treatment for VZV infection [102]. Other treatment strategies include the administration of valacyclovir, famciclovir, or brivudin [103,104,105].

#### 2.3.4. EBV Infection and Burn Wounds

Regarding other *Herpesviridae* infections within burn wounds, an infection with Epstein–Barr virus (EBV) in pediatric burn patients was reported by Linnemann and MacMillan in 1981 [18]. Among 27 examined patients, only three children presented increased EBV antibody titers with no significant clinical manifestations. However, according to the authors’ knowledge, no other studies showed the presence of EBV infection in burn patients specifically.

## 3. *Parapoxvirus* Infection in a Burn Injury and the Presence of a Skin Graft

*Parapoxvirus* is a virus that belongs to the *Poxviridae* family, constituting a group of relatively large, DNA-containing viruses that are responsible for highly contagious zoonotic infections. Human infections by the orf virus are mainly due to contact with farm animals and infected fomites [106]. There are some reports of the orf virus infections induced by infected fomites in burn patients; an increased risk of orf virus infection also concerns patients with skin grafts [107]. Hsu et al., in 2016, reported a case of a patient with burn injuries affecting 35% of the TBSA who received skin grafts [108]. The patient was reported to have both direct and indirect contact with farm animals that could have been the source of infection. The immunohistochemical staining was positive for *Parapoxvirus* and negative for *Orthopoxvirus*. Additionally, the presence of the skin grafts, along with the areas of the burn wound, significantly affected the patient’s susceptibility to viral infection. To the best of the authors’ knowledge, this is the first reported case of orf infection in the case of a patient with burn wounds at the skin graft harvest site.

Orf virus affects epidermal keratinocytes in areas that are more immunocompetent than the physiological, intact skin barrier. The pathogenicity of orf virus is associated with vascular endothelial growth factor (VEGF) that promotes angiogenesis, facilitating the infection [109]. Viral VEGF is also believed to play a role in the formation of scabs, which is a typical characteristic associated with the healing of orf virus lesions; viral VEGF is a crucial factor in both the transmission and replication of orf virus [110]. It was suggested that immunosuppression due to burn wounds is associated with the upregulation of viral VEGF but, nevertheless, the exact mechanism of this process is not yet deciphered. In cases of burn patients, the exact etiology of orf virus infection may be quite unclear since orf virus may be transmitted by several vectors, including infected animals and fomites, or even by direct transmission during the grafting process. Thus, an exact and accurate examination, along with the medical history and review, should be provided.

### Orf Virus Detection and Treatment

Laboratory testing for orf virus includes cell culture isolation, ELISA, western blotting, electron microscopy, and restriction fragment length polymorphism [111,112]. Even among a wide range of available diagnostic methods, PCR remains the most common technique used for the detection of orf virus, providing 100% sensitivity and specificity estimated to be 93% [113]. Various therapeutic modalities were reported to be efficient regarding the treatment of orf virus infection, including cryotherapy, electrocautery, imiquimod, or cidofovir administration [114,115]. Usually, large orf lesions require excision and skin grafting [116].

## 4. A Rare Case of *Papillomavirus* Infection

Human papilloma virus (HPV) belongs to the *Papovaviridae* family of the DNA viruses. To date, 174 HPV subtypes have been described but, nevertheless, new species are continuously being detected [117]. HPV is primarily responsible for intraepithelial neoplasias on the skin as well as within the mucosal cells [118]. The major HPV transmission route includes sexual intercourse but, nonetheless, other factors, such as smoking, defective immune responses, or even deficiency of some vitamins or minerals, might also contribute to this process [119]. HPV replication might occur within areas with defective immune responses, which also include burn injuries where immune cells and responses are significantly impaired. Moreover, due to the rapid destruction of the skin layers, HPV replication may occur at even increased levels, usually leading to more severe clinical outcomes. In 1996, Camilleri and Milner reported a case of a 4-year old boy with a burn that included only a small surface on the left ring finger [120]. After approximately 4 weeks, the patient reported the presence of a “keloid scar” within the area of the previous burn. Because the basal layer of the skin remained intact, HPV was able to survive and replicate within the region of the burn wound. According to the authors, such remains of a burn injury may be hard to distinguish from hypergranulation tissue and, therefore, the accurate diagnosis of HPV infection may be misleading in some cases. Furthermore, one of the limitations is that the authors did not exactly explain the methods of the HPV detection and diagnosis. The only information provided was that histological examination should be performed to obtain reliable data. Nevertheless, it was the first reported case of HPV presence within the area of a burn wound.

## 5. Human Immunodeficiency Virus

Human immunodeficiency virus (HIV) constitutes a member of the *Lentivirus* genus of the *Retroviridae* family. HIV is divided into two major types—HIV type 1 and HIV type 2. Infection by HIV-1 is considered to be the main cause of acquired immune deficiency syndrome (AIDS), whereas infection by HIV-2 is more restricted to specific regions in the world, such as Africa, but it can also induce AIDS [121]. HIV infection induces mononucleosis-like syndrome within approximately 28 days of the virus contraction [122,123]. Later, a significant decrease in the number of CD4 cells and an increase in viremia develops in untreated patients [124]. Eventually, impaired immune responses and defective functions of the immune cells may lead to the development of chronic multiorgan diseases and periods of prolonged latency, as well as usually severe impairments within the central nervous system [125,126].

What is crucial regarding the clinical course of burn wounds are the patient’s age and sex, other comorbidities and injuries, as well as the TBSA of the burn. According to Edge et al. (2001), HIV-positive patients who suffer from a burn injury and do not present any stigmata of AIDS should be treated similarly to HIV-negative patients [127]. In the 1-year study that was performed in the Burns Unit at Queen Elizabeth Central Hospital (QECH), Blantyre, Malawi, out of the 342 patients who were included in the study, 40 of them (11.7%) were HIV positive [128]. Among those 40 HIV-positive patients, 85% were older than 16 years old. The majority of deaths among burn patients were due to sepsis and multiorgan dysfunction. Death due to complications of sepsis affected 53% of the HIV-positive patients and only 21% of the HIV-negative patients. HIV-positive patients had significantly lower CD4 counts (mean 383 mm^3^) compared to the HIV-negative patients (mean 937 mm^3^); thus, the patients’ HIV status was an independent predictor of the CD4 counts. Therefore, immune deficiency due to both HIV infection and the presence of a burn wound itself might have contributed to the increased mortality rate of patients. Likewise, HIV infection and HIV-related multiorgan dysfunction, along with impaired immune responses, in burn patients resulted in higher mortality rates. Nevertheless, those patients whose burn wounds were more than 30% of the TBSA experienced 100% mortality regardless of HIV infection status. Microbiological tests showed no differences in the bacterial cultures in HIV-positive or HIV-negative patients. The most common bacteria included *Escherichia coli*, *Staphylococcus aureus*, and *Pseudomonas aeruginosa*. Furthermore, the higher burn percentage of the TBSA was in direct proportion to the lower total number of the white blood cells, as well as neutropenia. Interestingly, in other studies, there was no difference between the HIV-positive and HIV-negative patients in terms of the mortality and morbidity rates [127,129]. HIV-positive patients are more prone to sepsis and sepsis-related death not only because of the impaired immune responses but also as a consequence of the presence of a burn injury itself, which further facilitates the immunosuppression. Therefore, the size of the burn’s TBSA constitutes an important factor predicting the clinical outcome of both HIV-positive and HIV-negative patients.

Mzezewa et al., in 2003, aimed to investigate the impact of HIV infection on the clinical outcome of patients with burn wounds compared to HIV-negative patients with burns [130]. The TBSA of the group of patients with burn wounds was within the range of 10–20%; the control group included a group of 13 non-burnt, HIV-negative patients and 15 non-burnt and HIV-positive patients. Twenty-eight percent of all of the burn patients turned out to be HIV positive. Microbiological tests showed the highest prevalence of *Staphylococcus aureus* and *Pseudomonas aeruginosa* from the biological material obtained from the burn wound. Hemoglobin and hematocrit levels were significantly lowered in HIV-positive burn patients in comparison to the control groups. Besides, the level of leukocytes and serum proteins was increased in the case of burn patients. HIV-positive burn patients also showed lower levels of CD4+ lymphocytes and overall CD4+/CD8+ ratio. The decreased level of CD4+ in HIV-positive patients was induced by several mechanisms, including cytotoxicity, apoptosis, syncytium formation, or autophagy [131]. Interestingly, IL-2, IL-6, and TNF-α levels were decreased, whilst IL-4 levels were increased in the group of HIV-positive, non-burnt volunteers in comparison to HIV-positive patients with burns; besides, both of the abovementioned groups showed a significant decrease in the INF-γ levels. The presence of a burn wound, along with a coexisting HIV infection, leads to the significant impairment of the immune cells (especially T lymphocytes) and, thus, also a defective release of various cytokines; this mechanism also explains the depletion of the CD4+ lymphocytes [132]. Among 1217 burn patients, the prevalence of HIV infection was equal to 0.5% (*n* = 5), who were HIV positive in the cross-sectional study performed by Salehi et al., in 2015 [133]. In this study, all of the HIV-positive patients were males with a mean age of 39 years and had relatively extensive burn areas. HIV-positive patients presented a significantly longer duration of the hospitalization course. However, the mortality rate did not differ statistically in HIV-positive or HIV-negative groups. The number of HIV infections in burn patients significantly differs between nations, mainly because of the various socioeconomic statuses and medical care standards, which result in a different susceptibility to viral, bacterial, and fungal infections.

Blood transfusions may also constitute potential sources of viral infections, including hepatitis B virus (HBV), hepatitis C virus (HCV), or HIV type 1 and 2 [134,135]. Even though HIV type 2 presents lower pathogenicity and frequency in blood for transfusions, incidents of HIV type 2 seropositivity in blood donor samples are still possible [136]. There is a need for blood transfusions, especially among severely burned patients, since they tend to experience anemia throughout their hospitalization [137,138]. Therefore, usually, large amounts of blood are required to prevent impaired erythropoiesis and serious blood loss. Other factors, such as a burn greater than 20% of the TBSA, a low BMI status, and abnormal white blood cell counts, might be potential indicators during consideration for blood transfusion [139]. Tavousi et al. showed that, in a group of 701 burn patients, the mortality rate was two times higher among patients who had received blood transfusions compared to groups that were not treated with any blood products [140]. To date, there is no strategy to prevent HIV transmissions during blood perfusions [137]. Even though the risk of the HIV infection via blood transfusion is still probable, the seroprevalence of HIV among blood donors showed a significant decline, especially from 2004 to 2016 [141].

## 6. Conclusions

Patients with burn wounds present significantly higher susceptibility to a range of infections, including not only common bacterial and fungal infections, but also viral ones [142,143]. This is mainly due to the impaired immune responses and defective release of cytokines, an inappropriate vascular organization within the area of a burn injury, intensification of severe oxidative stress, or the presence of necrotic tissues that constitute the potential area of pathogenic growth or reactivation [144]. Viral infections in burn patients may be either primary or reactivated from their latency state. Immunosuppression results in a significant amount of reactivations of latent viral infections. It should be considered that both immunosuppression and prolonged hospitalization may significantly affect susceptibility to viral infections. Moreover, besides common viruses like HSV or CMV, some infections might be induced by less common ones such as orf virus or EBV. What should be considered while evaluating the published works regarding viral infections in burn patients is that some of the results are contradictory, for example, in some of the reports, particular viral infections are considered to affect the morbidity and mortality rates, whereas in other articles, no significant statistical correlation was proven. However, more research should be done to maximize the accuracy of viral infection diagnosis and, thus, the quickest introduction of the proper treatment therapy.

## Supplementary Materials

The following are available online at https://www.mdpi.com/1999-4915/12/11/1315/s1, Table S1: An overview of the published reports regarding viral infections in burn wounds.
