# Supplementary material for: Viral Infections in Burn Patients: A State-Of-The-Art Review
Source: Viruses. 2020 Nov 17;12(11):1315. doi: 10.3390/v12111315 (PMC7698518; doi:10.3390/v12111315)
Supplement: Supplementary file 1 [file viruses-12-01315-s001.pdf]

Supplementary Materials: Table S1: An overview of the published reports regarding viral infections in burn wounds.

Authors	Journal	Year of publication	Study type	Overall number of patients	Number of patients with viral infections	Age of patients (mean or range)	Sex (F – Female, M – male)	Total body surface area of burn (mean)	Viral infections	Other infections	Clinical manifestations
Wurzer et al. [31]	Burns	2017	Retrospective analysis (original study)	613	28	ND	F: 247<br>M: 366	38%	HSV-1<br>VZV<br>EBV<br>CMV	*P. aeruginosa*	Pneumonia<br>Sepsis
Sen et al. [34]	Journal of Burn Care & Research	2012	Retrospective analysis (original study)	71	21	39.7 years (mean)	ND	45.9%	HSV	*A. baumannii*<br>*P. aeruginosa*<br>*E. coli*<br>*C. albicans*<br>*Yeast*<br>*Enterococcus sp.*<br>*Methicillin-resistant S. aureus*<br>*S. aureus*<br>*S. pneumoniae*<br>*Corynebacterium*<br>*E. cloacae*<br>*H. influenzae*<br>*S. viridans*<br>*S. marscencs*<br>*Fusarium sp.*<br>*Mould*<br>*E. aerogenes*<br>*Stenotrophomonas*<br>*Bacillus*<br>*Curvularia sp.*<br>*C.*	Cutaneous HSV lesions<br>Respiratory infections

										guillermondii<br>*Paecilomyces*<br>*Aspergillus*	
Roberts et al. [44]	Journal of Burn Care & Research	2013	Prospective review (Original study)	48	10	17-80 years (range)	F: 2*<br>M: 8*	ND	HSV-1	ND	Facial herpetic lesions<br>Fever<br>Pain around mouth
Peppercorn et al. [38]	Journal of Burn Care & Research	2010	Case report	1	1	58 years (mean)	M: 1	68%	HSV-2	*A. flavus*<br>*E. cloacae*	Fever<br>Corneal opacification and erosion<br>Gastrointestinal bleeding<br>Hypotension<br>Acute renal failure<br>Atrial fibrillation
McGill & Cartotto [26]	Burns	2000	Case report	1	1	10 months	F: 1	12%	HSV-1	*S. aureus*<br>B hemolytic *Streptococci*<br>*H. influenzae*<br>*P. aeruginosa*	Fever<br>Pneumonia<br>Vesicles and vesicopustules on the right thigh, forearm, cheek and left lower eyelid<br>Tongue ulceration
Sobouti et al. [32]	International Journal of Burns and Trauma	2018	Case report	1	1	1 year	M: 1	0.5%	HSV	ND	Blisters, vesicles and erythema within burn wound

Fidler et al. [36]	The Journal of Trauma Injury, Infection and Critical Care	2002	Retrospective study (original study)	95	14	44 years (mean)*	ND	≥ 20%	HSV-1	ND	Facial rashes
Cook et al. [37]	Journal of Burn Care & Research	2017	Case report	1	1	58 years	F: 1	40%	HSV	*E. faecalis*<br>*P. aeruginosa*<br>Candidiasis<br>*Mucor*	Acute kidney injury<br>Acute tubular necrosis<br>Pneumonia<br>Hepatitis<br>Massive liver necrosis<br>Intracranial hemorrhage within the left basal ganglia<br>Intraperitoneal and retroperitoneal hematoma
Bourdarias et al. [22]	Burns	1996	Original study	11	11	39 years (mean)	F: 2<br>M: 9	22.9%	HSV-1	ND	Hyperthermia
Bordes et al. [39]	Burns	2009	Case report	1	1	43 years	M: 1	65%	HSV-1	*P. aeruginosa*<br>Candidiasis	Sepsis<br>Septic shock<br>Pneumonia<br>Encephalitis
Werdin et al. [40]	Journal of Clinical Microbiology	2008	Case report	1	1	23 years	M: 1	32%	HSV-1<br>CMV<br>VZV	*S. epidermidis*	Fever
Gong et al. [64]	Acta Biochimica et Biophysica Sinica	2013	Original study	160	108	32.3 years (mean)	F: 42<br>M: 66	35.3%	CMV	ND	ND

Gibbs et al. [63]	Journal of Burn Care & Research	2015	Case report	1	1	41 years	M: 1	72%	CMV	ND	colitis
Bordes et al. [60]	Burns	2010	Prospective study (original study)	29	15	63 years (mean)	F: 7<br>M: 22	≥ 15%	CMV	Coagulase-negative *Staphylococcus*	Pneumonia<br>Central venous catheters-related infections<br>Urinary catheter-related infections<br>Cholecystitis
Hamprecht et al. [62]	Journal of Clinical Microbiology	2005	Case report	1	1	40 years	F: 1	65%	CMV	*S. epidermidis*<br>*S. aureus*	Pneumonia
Hsu et al. [67]	Journal of Infectious Diseases	2016	Case report	1	1	4 years	F: 1	35%	Orf Virus	ND	ND
Camilleri & Milner [74]	Burns	1996	Case report	1	1	4 years	M: 1	ND	HPV	ND	Keloid scar
Salehi et al. [84]	International Wound Journal	2015	Original study	969	5	39.4 years (mean)	M: 5*	29.3%*	HIV	*S. aureus*<br>*C. perfringens*	ND
Mzezewa et al. [81]	The British Association of Plastic Surgeons	2003	Prospective study (original study)	54	15	15-45 years (range)	F: 5*<br>M: 10*	10-20% (range)	HIV	*S. aureus*<br>*P. aeruginosa*	ND
James et al. [79]	Burns	2003	Original study	342	40	Age ≤ 15: 5.2 years (mean)<br><br>Age > 16: 32.4	Age ≤ 15:<br>F: 122<br>M: 109<br><br>Age > 16:	Age ≤ 15: 13%<br>Age > 16: 15%	HIV	*E. coli*<br>*S. aureus*<br>*P. aeruginosa*<br>*S. paratyphi*	Sepsis<br>Hypovolemic shock<br>Diarrhoea<br>Tetanus<br>Pneumonia

						years (mean)	F: 47 M: 64				Respiratory distress
Edge et al. [78]	Burns	2001	Original study	661	33	31.6 years (mean)*	F: 19* M: 14*	26%*	HIV	ND	Pneumonia Renal failure Septicaemia Tuberculosis

F – Female; M – Male; ND – No Data; HSV-1 – Herpes Simplex Virus 1; VZV – Vericella Zoster Virus; EBV – Epstein-Barr Virus; CMV – Cytomegalovirus; HSV – Herpes Simplex Virus; HSV-2 – Herpes Simplex Virus 2; HPV – Human Papilloma Virus; HIV – Human Immunodeficiency Virus; * - among viral infected patients
